# Thymus development and infant and child mortality in rural Bangladesh

**DOI:** 10.1093/ije/dyt232

**Published:** 2013-12-22

**Authors:** Sophie E Moore, Anthony JC Fulford, Yukiko Wagatsuma, Lars Å Persson, Shams E Arifeen, Andrew M Prentice

**Affiliations:** ^1^MRC International Nutrition Group, Department of Population Health, London School of Hygiene & Tropical Medicine, London, UK, ^2^Department of Clinical Trials and Clinical Epidemiology, Faculty of Medicine, University of Tsukuba, Tsukuba, Japan, ^3^International Maternal and Child Health, Department of Women's and Children's Health, Uppsala University, Uppsala, Sweden and ^4^International Centre for Diarrhoeal Disease Research, Bangladesh (ICDDR,B), Dhaka, Bangladesh

**Keywords:** Immune function, nutrition, pregnancy, thymus

## Abstract

**Background** Data from West Africa indicate that a small thymus at birth and at 6 months of age is a strong and independent risk factor for infection-related mortality up to 24 and 36 months of age, respectively. We investigated the association between thymus size (thymic index, TI) in infancy and subsequent infant and child survival in a contemporary South Asian population.

**Methods** The study focused on the follow-up of a randomized trial of prenatal nutritional interventions in rural Bangladesh (ISRCTN16581394), with TI measured longitudinally in infancy (at birth and weeks 8, 24 and 52 of age) and accurate recording of mortality up to 5 years of age.

**Results** A total of 3267 infants were born into the Maternal and Infant Nutrition Interventions, Matlab study; data on TI were available for 1168 infants at birth, increasing to 2094 infants by 52 weeks of age. TI in relation to body size was largest at birth, decreasing through infancy. For infants with at least one measure of TI available, there were a total of 99 deaths up to the age of 5 years. No association was observed between TI and subsequent mortality when TI was measured at birth. However, an association with mortality was observed with TI at 8 weeks of age [odds ratio (OR) for change in mortality risk associated with 1 standard deviation change in TI: all deaths: OR = 0.64, 95% confidence interval (CI) 0.41, 0.98; *P* = 0.038; and infection-related deaths only: OR = 0.32, 95% CI 0.14, 0.74; *P* = 0.008]. For TI when measured at 24 and 52 weeks of age, the numbers of infection-related deaths were too few (3 and 1, respectively) for any meaningful association to be observed.

**Conclusion** These results confirm that thymus size in early infancy predicts subsequent survival in a lower mortality setting than West Africa. The absence of an effect at birth and its appearance at 8 weeks of age suggests early postnatal influences such as breast milk trophic factors.

## Introduction

The past two decades have seen a growing interest in the influence of the early life environment on early development and long-term health outcomes; the ‘Developmental Origins of Health and Disease’ (DOHaD) hypothesis. The majority of published data within this field of research have focused on chronic disease outcomes, with only limited data on the developmental origins of immunity and infectious disease risk or in resource-poor settings where this would be most relevant. Evidence to support the concept of early immune programming comes from studies on vaccine responses^1^^,^^2^ and on the impact of the early life environment on thymus development^3^^,^^4^ and function.^5^^,^^6^. Given the central role of the human thymus in immune development, understanding whether and how it can be shaped in early foetal and postnatal life, and the long-term impact of this, is important.

The human thymus is a primary lymphoid organ where bone marrow-derived precursors undergo differentiation and selection, ultimately leading to the migration of positively selected thymocytes to the periphery.^7^^,^^8^ The early life development of the thymic micro-environment is therefore critical for the establishment of a normal peripheral T-lymphocyte immune system. Thymus development commences early during fetal life, with the most critical period of growth thought to occur between 7 and 14 weeks of gestation.^9^ Although there remains lack of consensus over the pattern and timescale of ‘normal’ thymus growth and involution in humans,^10^ data suggest a substantial increase in growth over the first 6 months of postnatal life,^3^ followed by a temporary decrease until late infancy,^11^ with continuous growth thereafter until involution in adolescence.

Thymus development in infants can be assessed sonographically using a validated method in which the transverse diameter of the thymus and the sagittal area of its largest lobe are multiplied to give a volume-related thymic index (TI).^12^ Research from our group and others using this technique has shown that the development of the human thymus is critically sensitive to environmental exposures during early development.^3^^,^^4^^,^^13^ A limitation of these studies, however, is the lack of long-term follow-up to assess any possible consequence of these early life effects.

We have previously reported on the environmental determinants of thymic development in a large cohort of rural Bangladeshi infants.^4^ We summarize the key findings here: TI was strongly positively correlated to current infant size (weight or length) and significantly larger in male infants although, following adjustment for infant weight, these differences only remained significant for the measurements taken at 24 and 52 weeks of age. A significant effect of season of measurement was observed at all time points; the measurements taken at birth showed a biomodal pattern, with largest TIs observed when measured in June and December and smallest in March and September. At all other time points, TI peaked only once during the hot and dry months of the year, falling in size during the monsoon and winter months. TI was not associated with the prenatal supplementation groups, but offspring of women who had received counselling in exclusive breastfeeding had smaller TIs at 8 weeks of age; although, by 52 weeks of age, infants who had breastfed for greater than the median duration of days had a larger TI. Within this analysis we did not, however, look at the longer-term effects of thymic development on infant outcomes.

Evidence exists from both animal studies and observational research in humans to suggest poor thymus development may lie on the causal pathway between intra-uterine growth retardation (IUGR) and early mortality. Studies using rodent models show that low birthweight, resulting from maternal protein restriction during pregnancy, followed by catch-up growth in rodents, is associated with shortened lifespan, whereas protein restriction and slow growth during lactation lead to an increased lifespan.^14^ It is proposed that these effects are mediated through differential thymus growth.^15^ Research from Guinea-Bissau, West Africa, indicates that a small thymus at birth and at 6 months of age is a strong and independent risk factor for infection-related mortality up to 24 and 36 months of age, respectively.^16^^,^^17^ Given the central role of the thymus in the establishment of T-cell mediated immunity and the evidence to suggest a long-term effect of impaired early thymic development on subsequent mortality, but the lack of any further replication of this observation, we investigated the association between thymic index in infancy on subsequent infant and child mortality in a contemporary cohort from rural Bangladesh.

## Materials and Methods

### Study population

The study was conducted in the rural Matlab region of Bangladesh. This population is well characterized, having participated in a health and demographic surveillance system (HDSS) since 1963 as part of a health-related research programme led by the International Centre for Diarrhoeal Disease Research, Bangladesh (icddr,b). In 2001, icddr,b initiated the Maternal and Infant Nutrition Interventions, Matlab (MINIMat) study (ISRCTN16581394), randomizing all pregnant women in Matlab to receive a combination of protein-energy and micronutrient supplements. Full details of the MINIMat study are given elsewhere.^18^ In brief, on enrolment (around 9 weeks of gestation), women were randomly allocated to a prenatal food supplement [randomized to start early in the first trimester of pregnancy (early start) or in the second trimester (usual start), as per the existing national programme] in combination with three separate daily micronutrient supplements: [(i) the UNICEF/UNU/WHO preparation of 15 different micronutrients including 30 mg iron and 400 μg folic acid; (ii) 60 mg iron and 400 μg folic acid; or (iii) 30 mg iron and 400 μg folic acid] from week 14 during pregnancy. All supplements continued up to birth. Perinatally, women were further randomized to receive exclusive breastfeeding counselling or the standard health counselling provided by local caregivers. The prenatal arm of the study was completed in June 2004 with a total of 3267 singleton infants born.

### Method

Within the main MINIMat trial, we looked at the impact of early life nutritional and environmental determinants of infant thymic development. The main findings from this study have been published elsewhere.^4^ For this study on thymic development, mothers and their infants were recruited from the main MINIMat trial either at birth (for health centre deliveries) or at follow-up (for all other births).

For health centre deliveries, birthweight, length, head circumference and knee-heel lengths were measured immediately following delivery. For births occurring at home, a birth notification system was established to ensure that study staff were made aware of births as soon as they occurred. A female paramedic then visited the newborn within 72 h of birth. Birthweight was measured by electronic or beam scales, to the nearest 10 g. Recumbent length was measured using locally manufactured, collapsible length boards, precise to the nearest 1 mm.

Following delivery, infant weight and length were measured at monthly visits to 12 months of age, and then 3-monthly up to 2 years of age, then at 5 years of age, using regularly validated standard equipment. At separate monthly visits, a female fieldworker visited each household to collect information on infant feeding practices. Full details are described elsewhere.^18^ Data on feeding practices were later coded and breastfeeding status classified on the basis of current WHO recommendations^19^: (i) exclusive breastfeeding (breast milk only); (ii) predominant breastfeeding (breast milk plus other liquids such as water, tea or juice); and (iii) partial breastfeeding (other food or milk in addition to breast milk). Socio-economic status (SES) was estimated using a wealth index based on information on household assets and estimated by principal component analysis, producing a weighted score.^20^ Scores were grouped into quintiles.

### Thymus ultrasound

Thymus size was measured by trained paramedics using a portable ultrasound machine (Toshiba SSA 320A Justavision-200, Toshiba Medical Systems, Japan) together with a PVF-745V 5.0-7.0 MHz probe (Toshiba Medical Systems, UK). The TI was calculated using the mean of three measurements of both the transverse diameter and the sagittal plane.

A total of eight paramedics conducted the thymus scans. These paramedics had previously been trained to perform fetal biometry, to a high degree of accuracy.^21^ Prior to the start of the thymus scans, all sonographers received extensive training on the technique and underwent a series of standardization exercises to document intra- and interobserver reliability for both measurements (data not presented).

TI was assessed at four time points during infancy: within 24 h of birth and at 8, 24 and 52 weeks of age. Infants were scanned in the supine position, using a trans-sternal approach; sonography was not performed while the infant was crying, instead we would wait until the infant was calm before performing the scan. Only infants who were delivered at a health centre had a TI measurement made at birth. In order to maximize the number of data points at each subsequent visit, we then scanned all infants attending the clinic visits, irrespective of what other time points they had data from. Owing to a delay with the start of TI assessment, a large proportion of infants had passed 24 weeks by the time the study started; this resulted in more infants with TI measured at the final time point.

### Mortality data collection

The impact of nutritional supplementation on infant and childhood mortality is described in detail elsewhere.^18^ Ascertaining and attributing causes of death was done in accordance with the verbal autopsy standards that have been developed by the INDEPTH network and the World Health Organization.^22^ Community health research workers detected and recorded death through monthly household visits. Information was collected at home interviews with caretakers/relatives who had lived with the deceased in the same household at the time of death. Three independent physicians read the available information and assigned International Classification of Diseases, version 10 (ICD-10) codes. In case of disagreement, the cause was assigned after a consensus process. Definitions of mortality were: early neonatal—death within days 0–6 of birth; late neonatal—death between days 7 to 27; post neonatal—death between days 28 to 364; and child—death between days 365 to 1824. Further, an infant death was defined as all deaths of a live birth before 364 days of age, and a child death as death of a live birth before 5 years of age.

### Statistical methods

The analysis was performed in two stages: (i) adjusting TI measurements for season and infant size, sex and age; and (ii) logistic regression using the adjusted TI measurement as a predictor of death. TI measurements were adjusted by first regressing raw TI measurements on infant length, weight, age, sex and the first two Fourier terms for season of measurement.^4^ As previously, two separate seasonal patterns were calculated: one for birth data and the second for all postnatal time points, the latter fitted using a multilevel model. The standardized residuals were then calculated: the predicted value from the above regression was subtracted from each TI measurement and the result divided by the standard deviation for the time point. The resultant adjusted TI measurements were then entered as predictors in logistic regression analysis. A separate logistic regression predicting the risk of death between the age of measurement and 5-years models were fitted for each time point (birth, 2, 6 and 12 months of age). These logistic regression models were repeated for deaths due to infection by removing all data on children dying of other causes. Effects of breastfeeding status were explored by adding duration of exclusive breastfeeding to the logistic regression model. We also extended these models to include sex and its interaction with adjusted TI. Data were analysed using DataDesk (Version 6.1, Data Description, Ithaca, NY) and Stata Statistical Software: Release 12 (Statacorp, College Station, TX).

### Ethical considerations

The main MINIMat study and the thymus size addendum were approved by the Research Review and Ethical Review Committees, ICDDR,B, Dhaka, Bangladesh. The thymus size addendum was additionally approved by the Ethics Committee at the London School of Hygiene and Tropical Medicine. Written informed consent was obtained from all participating mothers following an explanation of the study in their own language.

## Results

**Figure 1:**
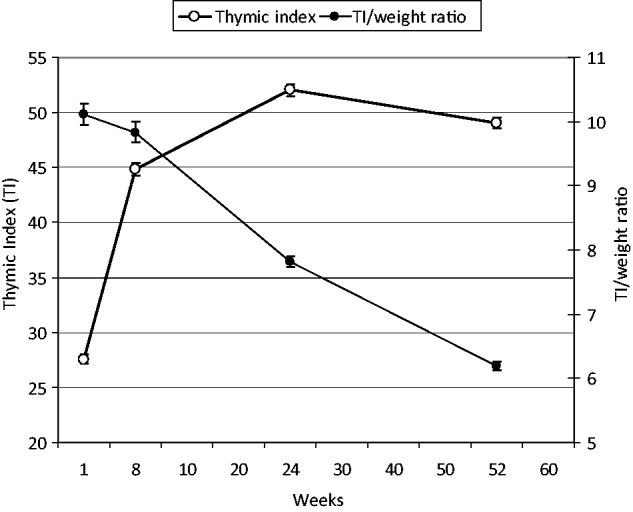
Changes in mean thymic index (open circles, thick line), and in mean thymic index expressed in relation to body weight (closed circles, thin line), with age. Data expressed as means and 95% confidence intervals

A total of 3267 singleton infants were born into the MINIMat study; full details of the main outcome measures are given in detail elsewhere.^18^ A total of 1168 infants had TI measurements taken at birth (health centre deliveries only), 1715 at 8 weeks, 1704 at 24 weeks and 2093 at 52 weeks of age; this increase to the final data collection point being a consequence of a delay starting ultrasound measurements in the field. The corresponding figures for those with anthropometry data available, and hence adjusted TI measurements, were 1168 at birth, 1627 at 8 weeks, 1595 at 24 weeks and 2022 at 52 weeks of age. Figure 1 details mean TI at birth (health centre deliveries only) and at 8, 24 and 52 weeks of infant age and TI expressed in relation to body weight. As previously reported in detail,^4^ TI in relation to body size was greatest at birth, decreasing to the measurements taken at 52 weeks of age.

Within the MINIMat study, there was a total of 111 deaths before the cohort all reached 5 years of age. Of these, 34 were early neonatal deaths (0–6 days), 13 late neonatal deaths (7–27 days), 27 post neonatal deaths (28–364 days); and 37 child deaths (365–1824 days). For the purpose of this analysis, only subjects with data on TI have been included; this reduced the total number of deaths to 49: 17 in infancy (<365 days) and the remaining 32 in childhood (365–1824 days). Table 1 summarizes child survival status and cause of death among those with TI observations at each time point. For example, of the 1168 infants with a TI measured at birth, 1140 were alive at follow-up to 5 years of age, 14 died in infancy and 14 died in childhood. Cause of death included 12 infections, 1 asphyxia, 1 malformation, 13 other and 1 death had an unknown cause.

**Table 1:** Number of infants with TI measurements available at each time point, and the status and cause of death of these infants, by time point

	Number of infants		
	Birth	8 weeks	24 weeks
Total number of TI observations	1168	1627	1595
Status			
Alive	1140	1601	1572
Death in infancy	14	7	2
Death in childhood	14	19	21
Cause of death^a^			
Infection	12	8	3
Asphyxia	1		
Preterm, LBW			
Malformation	1		
Other	13	17	19
Unknown	1	1	1

LBW, low birthweight.

^a^Cause-of-death groups are based on ICD10 codes.

Table 2A summarizes results of logistic regression of TI at each time point (birth and 8, 24 and 52 weeks of age) on infant and child survival. TI measurements are adjusted for infant size, sex and season of measurement and are presented for all deaths combined, then separately for infection-related deaths only. Analyses of the TI measurements taken at birth are also presented with neonatal deaths excluded. Odds ratios (OR) and 95% confidence intervals (95% CI) are presented for the change in mortality risk with a 1 standard deviation change in TI. TI when measured at birth was not associated with risk of death up to the age of 5 years. For measurements made at 8 weeks of age, a marginally significant association was observed between TI and death (OR = 0.64, 95% CI 0.41, 0.98; *P* = 0.038) which was strengthened when only infection-related deaths were included (OR = 0.27, 95% CI 0.14, 0.74; *P* = 0.008). For TI at 24 weeks of age, no association was observed in the model with all deaths included, although when infection-relation only deaths were included, a significant association appeared (OR = 0.27, 95% CI 0.08, 0.95, *P* = 0.041). However, this analysis was based on only three deaths, and so must be interpreted with caution. Only a single death occurred up to 5 years of age in subjects with a TI at measured at 52 weeks of age, and hence an analysis would not be meaningful. Controlling for breastfeeding made a negligible difference to the results (Table 2B). Finally, we tested for a sex-TI interaction, but no significant results were obtained (data not presented).

**Table 2:** (A) Logistic regression of thymic index on survival for all deaths, and infection-related deaths only. Table 2B Association controlling for duration exclusively breastfed

		A			B		
Time point	Deaths (number)	OR per SD^a^	95% CI for OR	*P*-value	OR per SD^a^	95% CI for OR	p-value
All deaths								
Birth	28	0.93	0.63, 1.36	0.69		0.92	0.62, 1.35	0.67
Birth (NN deaths excluded)	24	0.95	0.63, 1.44	0.82		0.95	0.63, 1.43	0.8
8 weeks	26	0.64	0.41, 0.98	**0.038**		0.63	0.41, 0.97	**0.035**
24 weeks	23	0.93	0.61, 1.40	0.73		0.92	0.61, 1.39	0.69
52 weeks	22	1.02	0.67, 1.55	0.94		1.01	0.66, 1.54	0.98
Infection-related deaths only^b^								
Birth	12	0.62	0.32, 1.19	0.15		0.61	0.32, 1.18	0.14
Birth (NN deaths excluded)	9	0.62	0.29, 1.30	0.20		0.62	0.29, 1.30	0.20
8	8	0.32	0.14, 0.74	**0.008**		0.31	0.13, 0.73	**0.007**
24	3	0.27	0.08, 0.95	**0.041**		0.29	0.08, 1.03	**0.056**

OR, odds ratio; SD, standard deviation; CI, confidence interval; NN, neonatal. Values in bold represent significant associations between thymic index and death, at the <0.05 level.

**^a^**OR for change in mortality risk associated with 1 SD change in thymic index.

^b^For infection-related deaths after 52 weeks of age, only one death was observed and hence analysis was not performed. Within the adjusted analysis, in no case was the breastfeeding term significant.

## Discussion

Previous studies from Guinea Bissau report associations between thymus size when measured at birth^16^ and at 6 months of age^17^ with subsequent mortality up to 24 and 36 months of age, respectively; infants with a smaller thymus had increased risk of subsequent mortality from infection-related causes. The current study, using identical techniques to measure thymus size as in Guinea Bissau, provides some further evidence to support an association with child survival to 5 years of age in a much larger cohort of infants from rural Bangladesh, with serial ultrasound measurement available from birth to 1 year of age. At 8 weeks of age, a significant trend was observed between TI and subsequent mortality from all causes and for infection-related deaths only.

Infectious diseases remain the major cause of morbidity and mortality in infants and children under 5 years of age in living in sub-Saharan Africa. It is well established that low birthweight and IUGR infants have increased risk of morbidity and mortality during infancy, although the mechanism(s) for these effects are not known. Given the central role of the thymus in the establishment of a normal peripheral T-lymphocyte immune system, replication of the findings from Guinea Bissau within other comparative cohorts is important.

The fetal T-cell (and B-cell) development begins at the end of the first trimester, with a rapid expansion of cell numbers through early childhood. This expansion involves the *de novo* production and export by the thymus of transitional T-cells (recent thymic emigrants; RTE), which then mature into naïve cells in the periphery. Insults during critical windows of development may therefore programme the structure and/or function of the thymus, permanently altering specific cell populations and leading to subsequent immune deficiencies. Such cellular changes would likely include a diminished output of T-lymphocytes, indicated by a reduction in the number of circulating naïve T-cells. Whereas studies of RTE dynamics in humans have been complicated by the lack of definitive markers of RTE status, markers such as T-cell receptor excision circles (TRECs) within cell populations have typically been used to assess thymic output.^23^ More recently, the use of surrogate markers of RTE status such as the surface molecules CD31 (platelet endothelial cell adhesion molecule-1)^24^ and PTK7 (protein tyrosine kinase 7)^25^ are being implicated as robust measures to assess thymus output. In a setting of high infectious exposure, defects in T-cell immunity would likely put an infant at a greater risk of disease, supporting the biological plausibility of a link between TI and subsequent mortality.

The data on thymus development collected within the MINIMat trial represent the largest published dataset of this nature to date, and the most detailed with longitudinal measures between birth and 52 weeks of age. Consistent with the literature, TI in relation to body size was largest at birth, decreasing thereafter. There was also a strong influence of season of measurement on TI, indicating plasticity of the developing thymus to environmental exposures. Strengths of the current study therefore include the large sample size, the prospective and longitudinal nature of the data collected and the detailed recording of date and cause of death. Limitations of the study include the lack of any further measures of thymic output or function, and the reliance on a sonographic estimation of thymic volume.

The main limitation of the current analysis, however, is the relatively low number of deaths after 6 months of age, precluding any meaningful analysis of the TI data collected after this time. This observation is the most likely explanation for a difference in findings between the current study and those previously published from Guinea Bissau. In the study from Guinea Bissau, where infants were measured at birth and followed to 24 months of age, the neonatal mortality rate (MR) was 39/1000, infant MR 117/1000 and the under-two MR 175/1000.^16^ In the second study from the same population area, where infants were measured at 6 months of age and followed to 36 months of age, there were 68 deaths from a population sample of 923 children.^17^ In the current study from rural Bangladesh, the early neonatal MR (deaths before 7 days of age) was 28/1000, the infant MR 42/1000 and the childhood MR (to 5 years of age) 49/1000; these rates reflect far fewer deaths than observed in Guinea Bissau, especially after the neonatal period. Moreover there was a smaller proportion of infection-related deaths in Bangladesh.

Of note, the absolute TIs from the current study appeared larger than those published from the Guinea Bissau studies^16^^,^^17^ and from other published data from The Gambia^3^ and Denmark.^11^^,^^13^ Whereas all studies used an identical technique for assessing the size of the thymus, differences in the absolute sizes measured could result from differences in equipment used (note that the studies from The Gambia, Guinea Bissau and Denmark all used the same machine with similar, although not identical, probes, whereas the study from Bangladesh used a different machine and probe), inter-observer differences across sites, or potentially from genuine differences between population groups. A cross-sectional study performed on two South American native populations (the Tsimane of Bolivia and the Pumé of Venezuela) showed that, despite nearly identical anthropometric trajectories, Tsimane infants had larger thymuses than Pumé infants at all ages.^26^ The authors conclude that these findings reveal a cross-cultural difference in thymus size that is not driven by nutritional factors. Pumé infants have smaller thymuses than Tsimane, and the authors describe how Pumé mothers are subjected to greater seasonal and environmental stressors, compared with the Tsimane; this combination of adverse prenatal factors is likely to negatively impact on fetal thymic development and contribute to smaller thymus size in Pumé newborns. Translating these data back to the current study, the smaller TI in infants from Guinea Bissau compared with Bangladesh may reflect a ‘read-out’ of a poorer environment and contribute to the differences observed between TI and subsequent mortality. However, the fact that TIs from healthy Danish infants track almost exactly to those from Bissau makes this explanation unlikely. A prospective study of thymus development across population sites is thus warranted, to help understand the patterns and sources of variation in postnatal thymic development in more detail. However, given that the current analysis is based on differences in TI within a population group, it is unlikely that any differences in absolute size of the thymus have contributed to the observed lack of a consistent association between TI and infant and child mortality.

We have previously observed, within this population of Bangladeshi infants, that a smaller leg length relative to head circumference, assessed sonographically *in utero* and characteristic of head-sparing growth restriction, was predictive of a lower TI postnatally,^27^ indicating that growth patterns typical of poor fetal nutrition are associated with poor thymic development. Added to the data from the current analysis and those from Guinea Bissau, these findings infer that appropriate nutrition early in pregnancy is important for the developing thymus and for prevention of infection-related mortality through infancy and beyond.

Infant feeding status has been shown as an important determinant of thymus size in early infancy, with breastfed Danish infants observed to have larger TIs compared with those who were bottle fed,^13^ potentially due to the interleukin-7 (IL-7) content in breast milk promoting thymic growth.^6^ However, within the current study, breastfeeding status, as assessed by mean duration of exclusive breastfeeding, did not alter the associations between TI and mortality as reported. Whereas it is possible that the method selected to adjust for breast-feeding status within the model was not sensitive enough, this finding does not support the premise that the observed association between TI and subsequent mortality was a reflection of differential infant feeding practices driving thymic development.

In summary, the current study provides support for the previously observed associations between thymus size in infancy and subsequent childhood mortality. The lack of a strong and consistent association across time points is most likely a consequence of the lower rates of mortality observed in rural Bangladesh. Our previous demonstration of a likely link between early life nutrition and TI,^27^ and the evidence provided here and elsewhere of an impact on mortality from infectious diseases, also have implications for populations with low rates of infection. Future work in this field should focus on understanding the aetiology of discordant thymic development (small thymus in relation to body size), and the longer-term immunological consequences of this.

## Funding

The MINIMat project has been financially supported by UNICEF, the Department for International Development (DfID), Swedish International Development Cooperation Agency (Sida), the United States Agency for International Development (USAID), the Japanese Society for the Promotion of Science (JSPS) and the Child Health and Nutrition Research Initiative (CHNRI). The sub-study of thymic development was supported by the UK Medical Research Council (core funding to the MRC International Nutrition Group; MC-A760-5QX00).
